# TiO_2_-Based Nanomaterials for Gas Sensing—Influence of Anatase and Rutile Contributions

**DOI:** 10.1186/s11671-017-1875-5

**Published:** 2017-02-06

**Authors:** K. Zakrzewska, M. Radecka

**Affiliations:** 1 Faculty of Computer Science, Electronics and Telecommunication, AGH University of Science and Technology, al. Mickiewicza 30, 30-059 Cracow, Poland; 2 Faculty of Materials Science and Ceramics, AGH University of Science and Technology, al. Mickiewicza 30, 30-059 Cracow, Poland

**Keywords:** Nanopowders, Anatase, Rutile, TiO_2_, TiO_2_-SnO_2_, Gas sensing

## Abstract

The paper deals with application of three nanomaterial systems: undoped TiO_2_, chromium-doped TiO_2_:Cr and TiO_2_-SnO_2_ synthesized by flame spray synthesis (FSS) technique for hydrogen sensing. The emphasis is put on the role of anatase and rutile polymorphic forms of TiO_2_ in enhancing sensitivity towards reducing gases. Anatase-to-rutile transformation is achieved by annealing of undoped TiO_2_ in air at 700 °C, specific Cr doping and modification with SnO_2_. Undoped TiO_2_ and TiO_2_-SnO_2_ exhibit n-type behaviour and while TiO_2_: 5 at.% Cr is a p-type semiconductor. X-ray diffraction (XRD) has been applied to determine anatase-to-rutile weight ratio as well as anatase and rutile crystal size. Scanning electron microscopy (SEM) and transmission electron microscopy (TEM) have been used to characterize the structure and morphological parameters. Optical reflectometry enabled to find and compare the band gaps *E*_g_ of anatase and rutile predominated compositions. Electrical properties, i.e. the electrical conductivity and values of constant phase element (CPE), have been established on the basis of impedance spectroscopy. Dynamic responses of the electrical resistance as a function of hydrogen concentration revealed that predominance of rutile in anatase/rutile mixture is beneficial for gas sensing. Partial transformation to rutile in all three material systems under study resulted in an increased sensitivity towards hydrogen. It is proposed that this effect can be explained in a similar way as in photocatalysis, i.e. by specific band alignment and electron transfer from rutile to anatase to facilitate oxygen preadsorption on the surface of anatase grains.

## Background

Anatase and rutile are the most frequently encountered polymorphic forms of titanium dioxide and usually co-exist for samples prepared in typical technologies such as sputtering [1–4], flame spray synthesis [4–7] and sol-gel [8]. It has been demonstrated that for the purposes of photocatalysis, a certain ratio of anatase to rutile, corresponding to anatase content extending from 40 to 80%, is desired as it causes a synergetic effect [9]. Some authors [10, 11] attribute this effect to the specific parameters of electronic structure such as electron affinity, work function and, in a consequence, a flat band potential (see Table 1) [12–14].

**Table 1:** Basic properties of the polymorphic forms of TiO_2_ after [12–14]

Property	Anatase	Rutile
Density (g/cm^3)^	3.894	4.250
Space group	I4_1_/amd	P4_2_/mnm
Symmetry	tetragonal	tetragonal
Lattice parameters (nm)	a = 0.3784 c = 0.9515	a = 0.4594 c = 0.2959
Molecules/cell	4	2
Volume/molecule (·10^−3^nm^3^)	34.061	31.2160
Average static dielectric constant	48	110–117
Band gap energy [eV]	3.2–3.26	3.02–3.05
Work function [eV]	5.1	4.9
Transformation temperature	700–900 °C	

In our opinion, it is highly probable that the phenomena observed in catalysts and gas sensors composed of anatase-rutile mixtures may have the same physical basis. Therefore, they may be interpreted in terms of complementary behaviour of both constituents towards oxygen adsorption and electron transfer to the surface due to differences in band alignment [10, 11, 15].

The advantages of using anatase or rutile in gas sensing have been discussed since decades. Rutile as the most stable form of TiO_2_ has been proved to be useful for hydrogen [16] and high temperature oxygen detection [17]. Applications of anatase in gas sensors [18–20] are inherently related to the advent of nanotechnology that, due to a substantial increase in surface-to-volume ratio, enables low temperature operation. Not only nanotubes [21] but other different and sometimes even exotic nanoforms have been tested as gas sensors [20].

In the past years, quite extensive research has been devoted to the mechanism and basic factors affecting the irreversible transformation from anatase to rutile [22–26]. In the case of bulk polycrystalline materials, temperature at which transformation takes place is of about 600 °C but its range can be much wider especially for nanomaterials. Transition from anatase to rutile is not instantaneous [25] because it involves a substantial structural reconstruction. The fundamental factors affecting the rate and temperature of transition are initial grain size, chemical surroundings and impurities. It has been demonstrated that this transformation can be achieved not only by annealing [25] or athermal illumination [22] but also by incorporation of aliovalent (Cr) dopant [5] as well as isovalent (Sn) additives [27]. From the thermodynamic point of view [24], rutile is the most stable of all polymorphic, macrocrystalline forms of TiO_2_ but the stability of anatase is particle-size dependent in the case of nanomaterials. It has been demonstrated in [23] that at particle diameters below ca. 14 nm, anatase is more stable than rutile.

Under experimental conditions discussed here, both anatase and rutile grains are present which allows to study their influence on gas-sensing properties of TiO_2_-based nanomaterials. The aim of this work is to show beneficial effect of contribution of both forms on the sensitivity to hydrogen at relatively low temperature (below 400 °C). Interpretation of this effect that takes into account electron transfer from rutile to anatase grains is proposed.

## Methods

For the purposes of this work, the following nanomaterial systems prepared by flame spray synthesis (FSS) were studied:

- undoped TiO_2_
- chromium-doped TiO_2_:Cr
- TiO_2_-SnO_2_ nanopowders

Titanium diisopropoxide bis (TDIP) and/or titanium isopropoxide (TTIP), chromium acetylacetonate and tetramethyltin were used as Ti, Cr and Sn precursors, respectively. The method and preparation conditions have been previously described in detail [6, 7, 28].

Selected samples were exposed to heat treatment at 700 °C in air.

X-ray diffraction (XRD) studies were carried out with X’Pert MPD Philips diffractometer in the Bragg–Brentano geometry. Weight percentage of rutile *f*_R_ and crystallite diameters of anatase *d*_A_ and rutile *d*_R_ were determined in a standard way.

Scanning electron microscopy (SEM) and transmission electron microscopy (TEM) were employed to get an insight into morphology of sample. NOVA NANO SEM 200 (FEI Europe Company) and HR-TEM FEI TECNAI TF 20 X-TWIN microscopes were used, respectively.

Impedance spectroscopy was performed with the Solartron system (1260 + 1294 dielectric interface). The experimental parameters and data acquisition were controlled by means of the FRA software. A frequency range of 1–10^6^ Hz was covered, with 10 mV amplitude. The impedance spectra were analyzed using the ZView software. An equivalent circuit comprising one or two parallel resistors and a constant phase element (CPE) was used in the fitting procedure.

Optical spectra of diffuse reflectance *R*_Diff_(*λ*) were acquired using a double beam Lambda 19 Perkin Elmer spectrophotometer equipped with an integrating sphere and operating within a wavelength range of 250–2000 nm. Calibration of reflectance spectra was performed using a SRS-99-010 Spectralon standard.

Gas-sensing measurements were performed for tablets prepared from nanopowders compressed at 25 MPa and then annealed at 400 °C and covered with planar silver electrodes. Dynamic changes in the electrical resistance response, $\frac{R}{R_0}$ or $\frac{R_0}{R}$, depending on the type of conductivity, were detected over low-to-medium concentrations of 50–3000 ppm H_2_ at a constant temperature chosen within the range of 250–400 °C. *R*_0_ denotes electrical resistance in air as a reference gas, and *R* is its value upon interaction with hydrogen. Homemade set-up described in [29] was used for gas-sensing measurements.

## Results

**Fig. 1:**
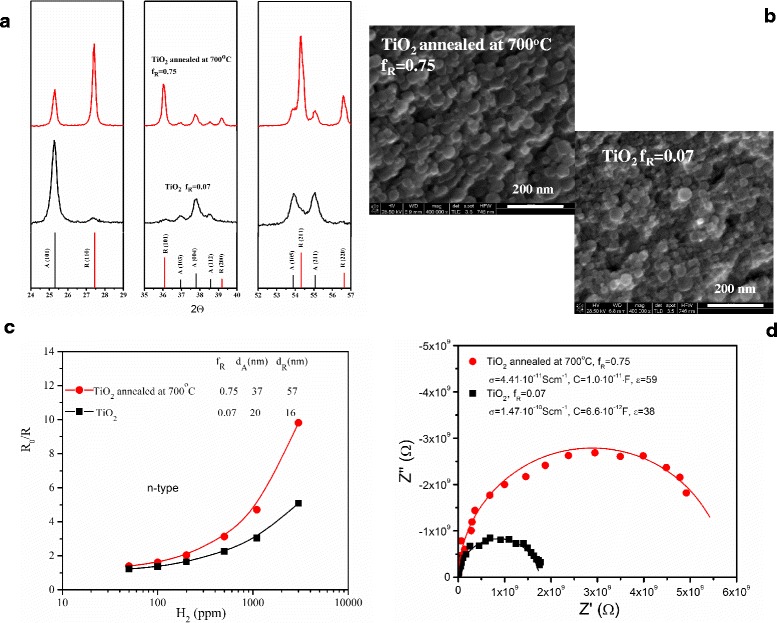
Comparison between anatase- and rutile-predominated undoped n-type TiO_2_ nanopowders obtained by flame spray synthesis (FSS): **a** X-ray diffraction patterns, **b** SEM images, **c** resistive-type response *R*_0_/*R* to hydrogen at 300 °C, **d** Nyquist plots recorded at 400 °C illustrating imaginary part of impedance *Z*″ as a function of its real part *Z*′: *f*_R_, rutile content; *d*_A_, diameter of anatase crystallite; *d*_R_, diameter of rutile crystallite; *R*_0_, electrical resistivity in the reference atmosphere (air); *R*, electrical resistivity under exposure to detected gas; *σ*, electrical conductivity; *C*, capacitance and *ε*, dielectric constant

Figure 1 illustrates the most important experimental results concerning the first nanomaterial system—undoped n-type TiO_2_. XRD (Fig. 1a) for as-prepared TiO_2_ nanopowder indicates the predominance of anatase polymorphic form. The content of rutile is low as determined from the analysis of this XRD pattern and amounts to *f*_R_ = 0.07. As expected, annealing of this powder at 700 °C created conditions favourable for the transformation of anatase to rutile as seen in Fig. 1a. However, as one can note, this transformation is incomplete. The sample is composed of rutile/anatase mixture with predominating rutile contribution (*f*_R_ = 0.75).

Annealing is accompanied by crystallite growth as determined from XRD pattern analysis and clearly seen in SEM micrographs presented in Fig. 1b. As found from XRD, both anatase and rutile crystallites increase their respective diameters from *d*_A_ = 20 nm, *d*_R_ = 16 nm (before annealing) to *d*_A_ = 37 nm, *d*_R_ = 57 nm (after annealing). However, spherical shape of nanograins is preserved with larger agglomeration due to annealing as concluded on the basis of SEM images.

This process has significant consequences as far as gas sensing (Fig. 1c) and electrical properties (Fig. 1d) are concerned. Figure 1c demonstrates the resistive-type response to hydrogen at 300 °C expressed in terms of the relative change of *R*_0_/*R*. This is suitable for n-type semiconductors as their electric resistance *R* decreases when reducing gas (hydrogen) is introduced. TiO_2_ annealed at 700 °C has better response than as-prepared sample.

On the other hand, a careful look into the electrical properties before exposure to the detected gas (Fig. 1d) indicates that the impedance increases considerably due to annealing. Parameters derived from the analysis of the impedance spectra: electrical conductivity *σ*, capacitance *C* and dielectric constant *ε* remain in accordance with the observed transformation from anatase to rutile.

**Fig. 2:**
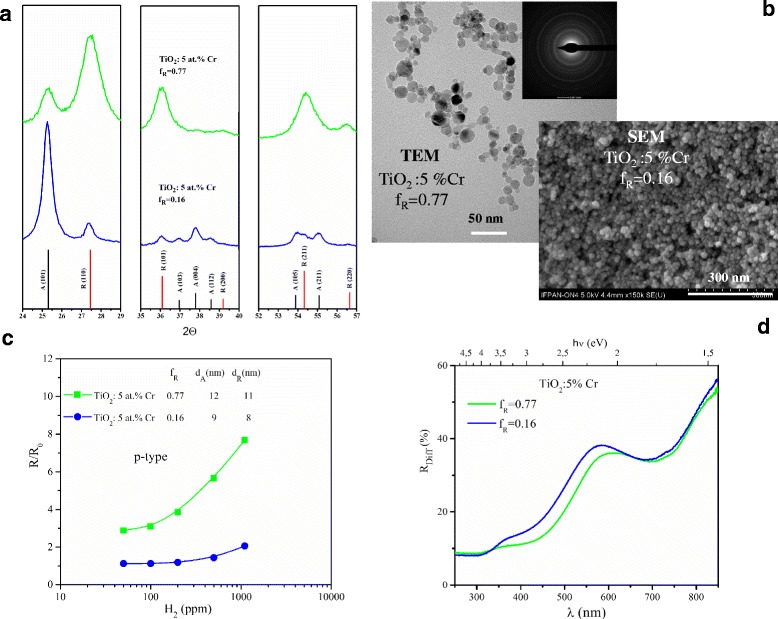
Comparison between anatase- and rutile-predominated p-type chromium Cr-doped TiO_2_ nanopowders obtained by flame spray synthesis (FSS): **a** X-ray diffraction patterns, **b** TEM/SEM images, **c** resistive-type response *R*/*R*_0_ to hydrogen at 300 °C, **d** diffused reflectance *R*_Diff_ as a function of wavelength *λ*; *f*_R_, rutile content; *d*_A_, diameter of anatase crystallite; *d*_R_, diameter of rutile crystallite; *R*_0_, electrical resistivity in the reference atmosphere (air); *R*, electrical resistivity under exposure to detected gas and hν, photon energy

Figure 2 summarizes the experimental results pertaining to the second system under study—Cr-doped p-type TiO_2_. Our intention was to contrast two samples with different anatase to rutile ratio with otherwise similar aspect, i.e. the same doping level and very close crystallite size. In order to obtain such different anatase to rutile ratio, slightly different technological procedures were employed [5, 6, 30]. In the case of anatase predominating sample, the total precursor mole number per minute was lower than that for rutile predominating sample which resulted in higher specific surface area SSA and smaller grain size as seen in Table 2. As mentioned in “Background” section, it is well known that anatase is stabilized when the crystallite diameter decreases. XRD patterns of TiO_2_: 5 at.% Cr for *f*_R_ = 0.16 and *f*_R_ = 0.77 clearly illustrate the predominance of either one (anatase) or another (rutile) polymorphic form as shown in Fig. 2a. Both anatase and rutile crystallites have comparable diameter for anatase dominated sample (*d*_A_ = 9 nm, *d*_R_ = 8 nm for *f*_R_ = 0.16). The same applies for rutile dominated one (*d*_A_ = 12 nm, *d*_R_ = 11 nm when *f*_R_ = 0.77). Morphology of both samples is quite similar as evidenced by SEM/TEM images (Fig. 2b).

**Table 2:** Summary of the experimental results of three different systems studied in this work

System	Sample	SSA (m^2^/g)	*f*_R_	*d*_A_ (nm)	*d*_R_ (nm)	*R*_0_/*R* at 300 °C, 3000 ppm H_2_	Figure
Undoped TiO_2_	As-obtained	67.7	0.07	20	16	4	1
	Annealed at 700 °C	–	0.75	37	57	10	
TiO_2_:5 at.% Cr	Mostly anatase	126.6	0.16	9	8	3	2
	Mostly rutile	102.9	0.77	12	11	12	
TiO_2_-SnO_2_	TiO_2_	57	0.05	25	9	2	3
	10% SnO_2_	60	0.73	28	14	8.5	

As chromium doping transfers conductivity of TiO_2_ to p-type, the definition of response has to be inverted as compared with n-type semiconductors because electrical resistance *R* increases upon hydrogen admission. Sample with higher rutile content exhibits visibly higher response in terms of *R*/*R*_0_ (Fig. 2c).

Different polymorphic phase composition finds its confirmation in optical properties of TiO_2_: 5 at.% Cr as well (Fig. 2d). As the band gap of rutile is by 0.2 eV smaller than that of anatase (see Table 1), its fundamental absorption edge in the spectral dependence of diffused reflectance shifts to longer wavelength. Additional absorption feature attributed to Cr band formation within the fundamental band gap of TiO_2_ is also displaced towards visible.

**Fig. 3:**
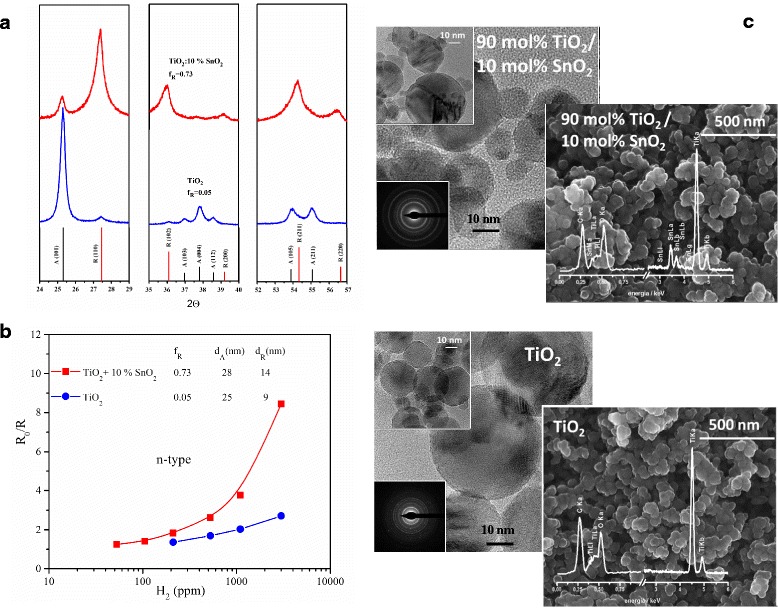
Comparison between anatase- and rutile-predominated n-type TiO_2_-SnO_2_ nanopowders obtained by flame spray synthesis (FSS): **a** X-ray diffraction patterns, **b** resistive-type response *R*_0_/*R* to hydrogen at 300 °C, **c** TEM and SEM images: *f*_R_, rutile content; *d*_A_, diameter of anatase crystallite; *d*_R_, diameter of rutile crystallite; *R*_0_, electrical resistivity in the reference atmosphere (air) and *R*, electrical resistivity under exposure to detected gas

Figure 3 resumes the most significant data for TiO_2_-SnO_2_ system under investigation. As seen in Fig. 3a, even a relatively small amount of SnO_2_ additive to TiO_2_ results in a dramatic reconstruction of crystallographic structure. Rutile contribution increases from *f*_R_ = 0.05 for pure TiO_2_ to *f*_R_ = 0.73 for TiO_2_: 10% SnO_2_. The crystallite size of anatase remains almost without changes (*d*_A_ = 25 nm for TiO_2_ and *d*_A_ = 28 nm for TiO_2_:10% SnO_2_). In contrast, diameter of rutile crystallites increases from *d*_R_ = 9 nm for TiO_2_ to *d*_R_ = 14 nm for TiO_2_:10% SnO_2_. No evidence of precipitation of SnO_2_ phase and detailed analysis of XRD patterns [28] indicates substitutional doping of Sn into TiO_2_ lattice.

As n-type conductivity is preserved upon incorporation of aliovalent additives, the sensor response is defined as *R*_0_/*R* in Fig. 3b. Much higher sensitivity is obtained in the case of TiO_2_:10% SnO_2_.

TEM and SEM images are given in Fig. 3c, for both TiO_2_ and TiO_2_:10% SnO_2_. Spherically shaped nanograins of TiO_2_ probably composed of smaller crystallites are clearly seen in TEM. SEM images reveal agglomeration process taking place.

## Discussion

**Fig. 4:**
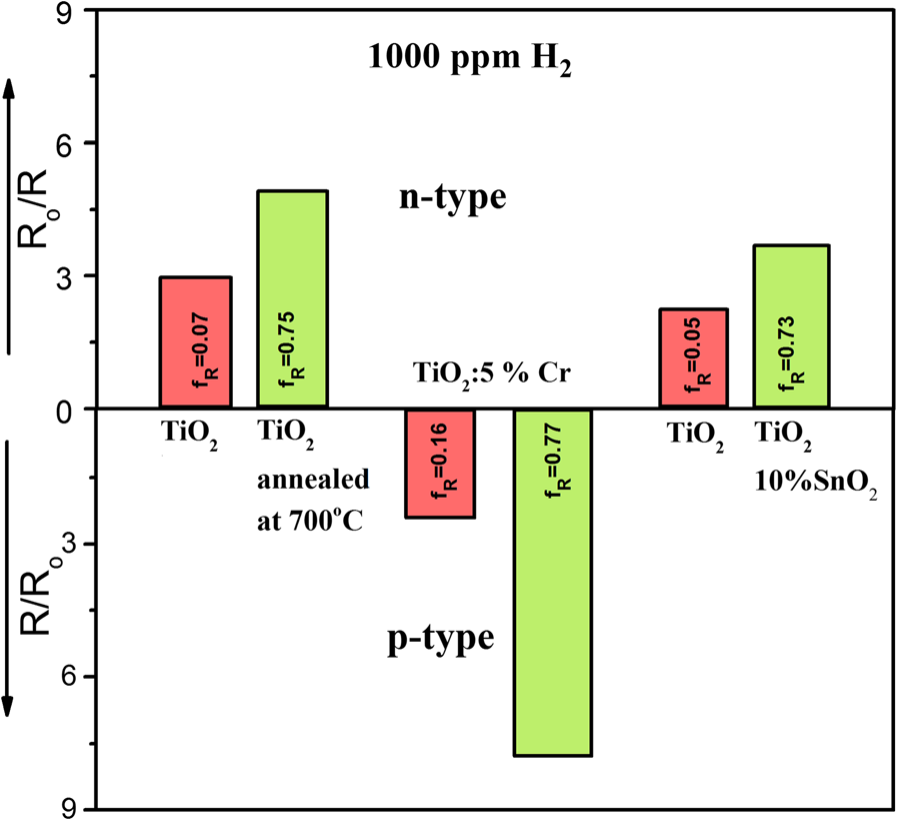
Comparison of resistive type responses towards 1000 ppm H_2_ at 300 °C for all TiO_2_ - based nanomaterial systems (n- and p-type) studied in this work; *f*_R_ rutile content

Recapitulation of the sensing performance of all material systems studied in this work is given in Fig. 4 for a fixed hydrogen concentration (1000 ppm H_2_) at a constant temperature of 300 °C. Two material systems, namely, undoped TiO_2_ and TiO_2_-SnO_2_ exhibit n-type response towards reducing gases, i.e. their electrical resistance decreases upon interaction with hydrogen. Only TiO_2_:5 at.% Cr behaves as a p-type semiconductor with its electrical resistance increase when reducing gas is introduced.

The reasons for such behaviour are very well known. Gas-solid interactions leading to the physical adsorption and chemisorption modify the electron/hole density in a relatively shallow region near the surface [31]. In the case of chemisorption of the reducing gas on the surface of an n-type semiconductor, a two-step interaction mechanism has been proposed [32]. The first step is considered to be an oxygen adsorption at the surface of the n-type semiconductor already exposed to the oxidizing atmosphere. In the second step, the surface reduction by the detected gas, e.g. hydrogen, methane and carbon oxide, takes place.

The chemisorption of oxygen may be described by the following reaction:

1 $$ {\mathrm{O}}_2+2{e}^{\hbox{'}}\to 2{\mathrm{O}}_{\mathrm{ads}}^{-} $$

This reaction results in a decrease in the surface conductivity. Upon exposure to a reducing gas such as H_2_, the following counter process takes place:

2 $$ {\mathrm{H}}_2+{\mathrm{O}}_{\mathrm{ads}}^{-}\to {\mathrm{H}}_2{\mathrm{O}}_{\mathrm{des}}+{e}^{\hbox{'}} $$

and electrons are reintroduced into the conduction band so that the surface conductivity is increased. As the reaction described by Eq. (2) is reversible, reducing gases in air can be detected by monitoring the change in the surface conductivity of metal oxides.

Eq. (2) is, in fact, an oversimplified description of the surface reaction, since it may also proceed via O_2_^−^ or O^−2^. The doubly charged oxygen ion is in general excluded from the considerations [33], because such a high charge on the ion may lead to instability, unless the adsorption site has a high Madelung potential. Higher reactivity of O^−^ as compared with O_2_^−^ makes the former one more probable [32, 33].

However, quite surprising conclusion can be drawn suggesting that rutile-dominated TiO_2_ nanomaterials exhibit higher sensitivity towards hydrogen than those with the prevailing anatase.

We propose to account for that in a similar way as it was done in the case of photocatalytic properties of TiO_2_. In fact, the fundamental phenomena such as surface oxidation in the first step of gas sensing have the same physical basic as in photocatalysis.

Synergetic effect in photocatalysis, discussed at the beginning of this work, is interpreted by some authors [9] as a result of specific electronic band alignment of anatase and rutile crystals leading to, for instance, successful space separation of photoexcited electron-hole pairs.

**Fig. 5:**
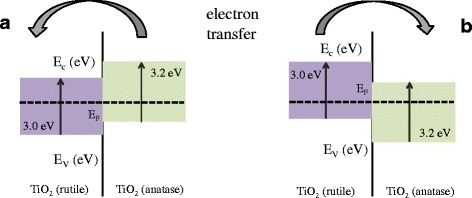
Two proposed valence and conduction band alignment mechanisms for the anatase-rutile mixture. **a** Electron injection from anatase to rutile. **b** Electron injection from rutile to anatase. The present study supports model **b**

Two opposite cases of band alignment presented in Fig. 5 reflect the state of the art of the studies on the electronic structure of anatase and rutile polymorphic forms of TiO_2_ [10, 11]. Historically, first was the model presented in Fig. 5a that has been derived from the measurements of flat band potential *V* ^0^_Fb_. Our own results of *V* ^0^_Fb_ determination [3] as a function of anatase content remain in accordance with the literature data [34–36]. It has been shown that *V* ^0^_Fb_ is by 0.2 more negative in anatase than in rutile which makes the conduction band minimum of rutile below that of anatase (Fig. 5a). In such a case, the electrons are injected to rutile. However, recent theoretical calculation by Scanlon et al. [10] and XPS studies [11] revealed that the opposite case is more probable, i.e. the conduction band minimum of rutile above that of anatase (Fig. 5b). Two arguments speak in favour of this picture. The first one is the difference in work function (5.1 eV for anatase and 4.9 for rutile, as shown in Table 1). The second one is related to the stability of rutile to adsorption of oxygen species [19] that makes the surface of anatase grains more active in the first step of gas sensing (Eq. 1).

In the proposed model of specific alignment of bands in rutile and anatase shown in Fig. 5b, the electrons are injected from rutile to anatase participating in more active oxidation reaction at the anatase grain surface. This phenomenon has been observed previously and used to explain significant improvement in gas sensing of TiO_2_-SnO_2_ nanocomposites [29]. Now, it seems that this mechanism can be successfully applied to rutile-anatase mixtures.

## Conclusions

The following nanomaterial systems prepared by flame spray synthesis (FSS) were studied:

- undoped TiO_2_
- chromium-doped TiO_2_:Cr
- TiO_2_-SnO_2_ nanopowders

Sample characterization was performed using standard methods such as XRD, SEM, TEM, impedance spectroscopy and optical reflectometry. In all cases, the crystallite size was below 60 nm which correlated very well with the possibility of gas-sensing measurements at relatively low temperatures of 300 °C. For each of the studied systems, we were able to discuss the cases of low and high rutile content. Moreover, n-type or p-type conductivity was observed. It turned out that rutile-dominated TiO_2_ nanomaterials exhibited higher sensitivity towards hydrogen than those with the prevailing anatase. This phenomenon could be accounted for in a similar way as in photocatalysis, i.e. by specific band alignment and electron transfer from rutile to anatase to facilitate oxygen preadsorption.

## Abbreviations

FSS: Flame spray synthesis
SEM: Scanning electron microscopy
TEM: Transmission electron microscopy
XRD: X-ray diffraction
